# Short-Term Effects of Nonnutritive Sweetener (Sucralose and Saccharin) Consumption on Glycemic Control and Gut Microbiota in Patients With Type 2 Diabetes: Protocol for a Double-Blind, Randomized, Placebo-Controlled, Crossover Trial

**DOI:** 10.2196/82695

**Published:** 2025-12-10

**Authors:** Huey Shin Tan, Harvinder Kaur Gilcharan Singh, Vanitha Mariappan, Nor Aini Jamil, Suhaili Naim Mustapa, Snigdha Misra

**Affiliations:** 1 Centre for Community Health Studies (ReaCH) Faculty of Health Sciences Universiti Kebangsaan Malaysia Kuala Lumpur Malaysia; 2 Center for Toxicology and Health Risk Studies (CORE) Faculty of Health Sciences Universiti Kebangsaan Malaysia Kuala Lumpur Malaysia; 3 Klinik Kesihatan Setapak Kuala Lumpur Malaysia; 4 Jeffrey Cheah School of Medicine and Health Sciences Monash University Malaysia Kuala Lumpur Malaysia

**Keywords:** saccharin, sucralose, nonnutritive sweetener, artificial sweetener, glycemic control, gut microbiota, type 2 diabetes mellitus

## Abstract

**Background:**

Nonnutritive sweeteners (NNSs) are widely used as sugar substitutes to help individuals with diabetes manage glycemic control. However, emerging evidence suggests that even low doses of NNSs, such as saccharin and sucralose, may adversely affect metabolic health by impairing glycemic regulation, potentially through alterations in the gut microbiota. In Malaysia, where gut microbiome research is still limited, particularly among individuals with type 2 diabetes mellitus (T2DM), further investigation is warranted to inform safe and evidence-based use of NNSs.

**Objective:**

This study aims to evaluate the short-term effects of saccharin and sucralose consumption on glycemic control and gut microbiota composition in adults with T2DM.

**Methods:**

This is a double-blind, randomized, placebo-controlled, crossover trial. A total of 33 adults with T2DM will consume sucralose (5 mg/kg body weight), saccharin (2 mg/kg body weight), or a placebo (calcium carbonate) in capsule form daily for 7 days per intervention arm, with a 4-week washout period. Data collection will include anthropometric measurements, biochemical assessments for glycemic control, dietary records, physical activity levels, and stool samples. The homeostatic model assessment for insulin resistance will be used to assess insulin sensitivity, while 16S rRNA V3-V4 region sequencing will be conducted to profile gut microbiota composition.

**Results:**

Recruitment is planned to begin in January 2026 and is expected to conclude by September 2026, with study completion anticipated by March 2027. As of December 2025, no participants have been enrolled.

**Conclusions:**

This trial will contribute novel insights into the effects of short-term NNS consumption on glycemic control and gut microbiota composition in individuals with T2DM. These findings may support evidence-based recommendations for NNS use in diabetes management and enhance understanding of microbiome-diet interactions in an ethnically diverse Asian population.

**Trial Registration:**

ClinicalTrials.gov NCT07124585; https://clinicaltrials.gov/study/NCT07124585

**International Registered Report Identifier (IRRID):**

PRR1-10.2196/82695

## Introduction

Nonnutritive sweeteners (NNSs) are widely used as sugar substitutes to reduce caloric intake and help manage blood glucose levels. Although previously considered metabolically inert, emerging evidence suggests that NNSs may influence host metabolism through their effects on the gut microbiota [1]. A landmark study in 2014 demonstrated that NNS-induced glucose intolerance in mice was mediated by gut microbiota alterations and was transferable to germfree mice via fecal microbiota transplantation [2]. However, subsequent studies have reported inconsistent findings, some indicating that NNSs alter microbial composition and impair glycemic control [3-6], while others have found no significant effects [7-9]. Variability in outcomes likely reflects differences in the type of sweetener, study design, host characteristics, and baseline microbiota composition.

Although all types of NNSs are categorized as sugar substitutes, they differ in their chemical structures, metabolism, and gut accessibility. Therefore, findings from one NNS cannot be generalized to all types of sweeteners. A recent study by Suez et al [3] in 2022 showed that while all tested NNSs altered the gut microbiome, only sucralose and saccharin significantly impaired glycemic responses [3]. These effects were reproduced in germfree mice colonized with microbiota from exposed individuals, strengthening the causal link between NNSs, gut microbiota, and metabolic outcomes. Therefore, this study focuses specifically on sucralose and saccharin.

Observational studies have also raised concerns that long-term consumption of sucralose and saccharin may lead to glycemic dysregulation, increased insulin resistance, or metabolic disturbances [2,3,10-13]. However, these effects have generally been observed at higher doses and with prolonged exposure. In contrast, this study will adopt a short-term, controlled intervention using doses well within the acceptable daily intake recommended by major health authorities, including the US Food and Drug Administration (FDA), the European Food Safety Authority, and the Joint FAO/WHO Expert Committee on Food Additives [14-17]. Both NNSs have been extensively evaluated and are considered safe for human consumption within their established acceptable daily intake limits.

To date, most NNS studies have focused on healthy populations, overlooking the fact that type 2 diabetes mellitus (T2DM) itself is associated with gut dysbiosis and altered metabolic signaling [18,19]. According to the International Diabetes Federation, an estimated 4.75 million adults in Malaysia were living with diabetes in 2024 [20]. The use of NNSs in Malaysia is expected to increase due to growing public awareness of the harmful effects of excessive sugar consumption and the implementation of a sugar tax on sugar-sweetened beverages. Consequently, the food industry has increasingly turned to NNSs for product reformulation.

Given these trends and the limited evidence among individuals with T2DM, this study aims to evaluate the short-term effects of sucralose and saccharin on glycemic control and gut microbiota composition in Malaysian adults with T2DM. Specifically, the primary objective is to assess changes in insulin sensitivity, while the secondary objective is to examine alterations in gut microbiota composition and diversity following exposure to different NNSs.

## Methods

### Study Design

**Figure 1:**
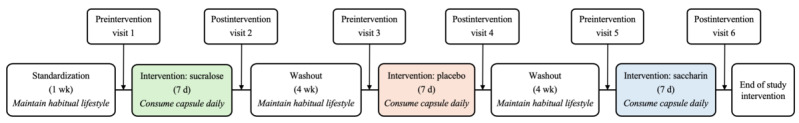
Overview of the study visits.

This study is a double-blind, randomized, placebo-controlled crossover trial. The crossover design allows each participant to serve as their own control across 3 intervention phases. Each participant will complete a 12-week protocol comprising a 1-week run-in period followed by 3 intervention phases, each separated by a 4-week washout period. A total of 6 study visits, comprising 3 preintervention and 3 postintervention assessments, will capture baseline and postintervention data. During the run-in and washout periods, participants will maintain their habitual diet and physical activity. The study will be conducted at a government primary care facility situated in an urban area. Recruitment will be facilitated through clinic listings and physician referrals. Written informed consent will be obtained after participants receive detailed verbal and written explanations of the study procedures, risks, and rights. The study timeline is summarized in Figure 1.

### Participants

This study aims to recruit a cohort of clinically stable patients representative of Malaysians with early-phase T2DM. Eligible participants will be males aged 30 to 59 years, with a BMI of 23.0 to 29.9 kg/m², classified as overweight [21]. Participants must have a clinical diagnosis of T2DM for 1 to 10 years, with baseline glycated hemoglobin (HbA_1c_) below 10%, and be managed with oral antidiabetic medications only. Individuals with stable, nonsevere chronic conditions, such as hypertension or dyslipidemia, will be included if they have maintained a consistent oral treatment regimen for at least 6 months before enrollment. All participants must have previously received dietary counseling for diabetes and agree to maintain their habitual diabetes-friendly diet and regular physical activity throughout the study. They must abstain from alcohol consumption during the intervention period and be literate in either English or Bahasa Malaysia to ensure full understanding of the study procedures and informed consent materials.

Exclusion criteria focus on lifestyle or dietary patterns that deviate from typical Malaysian practices or that could substantially alter the gut microbiota. Individuals will be excluded if they currently smoke or consume alcohol, have experienced more than 5% body weight change within the past 3 months, or have acute illness or major comorbidities, such as cardiovascular, psychological, neurological, renal, or endocrine disorders other than T2DM. Those with known intolerance or allergy to the test products, adherence to special diets (eg, vegetarian, ketogenic, or intermittent fasting), or use of medications or supplements (eg, probiotics) that may affect intestinal function or microbiota composition will also be excluded. Participation in another clinical trial within the past 3 months will preclude eligibility. Participants may withdraw voluntarily or be withdrawn for safety reasons, protocol deviation, or the need for antibiotic therapy. Diabetes management will continue as per clinical practice. Adverse events will be monitored and managed by the study physician following institutional protocols.

### Intervention

Participants will receive 1 of 3 interventions: sucralose, saccharin, or a placebo, for 7 consecutive days during each intervention phase. Participants will consume the assigned capsule each morning after breakfast with plain water to standardize timing and minimize missed doses. Dosages will be adjusted based on body weight according to national guidelines, with 5 mg/kg for sucralose and 2 mg/kg for saccharin [22]. The placebo, consisting of 500 mg of calcium carbonate, will be administered under identical conditions and is considered physiologically inert at this dose [9].

Randomization and allocation concealment will be conducted by external personnel not involved in recruitment, data collection, or analysis. A computer-generated block randomization sequence will assign participants to 1 of 3 intervention orders (sucralose, saccharin, or placebo) to ensure balanced allocation across participants. All test products will be encapsulated in identical opaque capsules and packaged in indistinguishable small plastic pouches. Labeling will be performed by external personnel according to the intervention sequence, where each package will be marked as “first intervention,” “second intervention,” or “third intervention” to maintain blinding. Both participants and investigators will remain blinded throughout the data collection period. The randomization code will be securely stored and accessible only in the event of a medical emergency.

### Outcomes

The primary outcome is the change in insulin sensitivity, assessed using the homeostatic model assessment for insulin resistance (HOMA-IR), derived from fasting glucose and insulin concentrations. The secondary outcome is the change in gut microbiota composition and diversity, analyzed through 16S rRNA gene sequencing. Alpha and beta diversity indexes will be calculated to evaluate within- and between-sample diversity, and differential abundance analysis will be performed to identify microbial taxa associated with each intervention.

### Sample Size

The sample size for this crossover trial was estimated based on detecting a medium effect size (Cohen *f*=0.25), consistent with findings from a recent meta-analysis of soluble fiber supplementation in T2DM, which reported moderate standardized mean differences for HOMA-IR (standardized mean difference −0.58, 95% CI −0.86 to −0.29) [23]. Using G*Power 3.1.9.6, we computed the required sample size for a within-subject repeated-measures ANOVA with 3 conditions (sucralose, saccharin, and placebo), assuming α=.05, and statistical power at 80%, and a moderate correlation (*r*= 0.5) among repeated measurements [24]. The analysis indicated that a minimum of 28 participants would be required. To account for potential dropout or noncompliance, we will recruit 33 participants.

### Ethical Considerations

This study was approved by the Universiti Kebangsaan Malaysia Research Ethics Committee (UKM/PPI/111/8/JEP-2025-322) on July 15, 2025. Procedures follow the Declaration of Helsinki and institutional ethics standards. This study is registered with ClinicalTrials.gov (NCT07124585), first released on August 8, 2025.

Written informed consent will be obtained from all participants before enrolment. Participants will receive a verbal explanation of the study and will be given adequate time to review the participant information sheet. An optional separate consent form will be provided for the storage and future use of deidentified biospecimens in ancillary studies.

Participant privacy and confidentiality will be protected throughout the study. Identifiable information will be collected only as necessary and will be stored separately from research data using unique participant codes. All datasets will be deidentified before analysis, and access to identifiable information will be restricted to authorized study personnel. No identifiable data or images will appear in publications or presentations.

Participants will receive financial compensation for time and transportation. Compensation for harm is not anticipated, as the interventions involve commercially available NNSs administered at doses considered safe. Any adverse events will be documented and managed according to institutional procedures, and medical care will be provided as needed.

### Data Collection

Data and biospecimens will be collected at baseline and after each intervention phase by trained research personnel as outlined in the study visit overview (Table 1).

**Table 1:** Overview of data and biospecimen collection by study visit.

		Screening	Intervention 1		Intervention 2		Intervention 3	
			Visit 1	Visit 2	Visit 3	Visit 4	Visit 5	Visit 6
**Anthropometry**								
	Weight	✓	✓	✓	✓	✓	✓	✓
	Height	✓						
	Waist circumference		✓		✓		✓	
	Body composition		✓	✓	✓	✓	✓	✓
	Blood pressure		✓	✓	✓	✓	✓	✓
**Biological specimens**								
	Blood		✓	✓	✓	✓	✓	✓
	Stool		✓	✓	✓	✓	✓	✓
**Questionnaire**								
	Sociodemographic and medical history	✓						
	Medication record	✓	✓		✓		✓	
	Dietary record	✓	✓	✓	✓	✓	✓	✓
	Physical activity record	✓	✓	✓	✓	✓	✓	✓
	Test product compliance record			✓		✓		✓
	Adverse effect reports			✓		✓		✓

Anthropometric measurements will be obtained using standardized procedures. Body weight will be measured using a calibrated digital scale with participants wearing light clothing, while height will be measured using a stadiometer, ensuring an upright posture and proper head alignment. The average of 2 readings will be recorded for both weight and height, and BMI will be calculated as weight in kilograms divided by height in meters squared (kg/m²). Waist circumference will be measured at the midpoint between the lowest rib and the iliac crest using a nonstretchable measuring tape [25]. Body composition, including body fat and muscle mass, will be assessed using bioelectrical impedance analysis following standard premeasurement guidelines [26]. Blood pressure will be measured twice after a 5-minute rest using an automated sphygmomanometer, and the mean of both readings will be recorded.

For biological specimens, blood samples of approximately 15 mL will be collected from participants at each study visit by trained nurses after a 30-minute rest period. Analyses will include fasting glucose, fasting insulin, glycated hemoglobin (HbA_1c_), lipid profile, and kidney function indicators. Insulin resistance will be estimated using the HOMA-IR, derived from fasting glucose and insulin levels [27]. Samples will be transported and processed according to biosafety and quality control procedures to ensure sample integrity for biochemical analysis and future molecular investigations. Stool samples of approximately 1 g will be collected using standardized fecal collection tubes provided to participants. Preintervention samples will be collected a day before each intervention phase, and postintervention samples will be obtained immediately after the final intervention day. Participants will receive detailed written and verbal instructions to ensure correct collection and handling procedures [28].

Sociodemographic and medical history information will be obtained through structured interviews and verified against medical records. Medication use will be reviewed at each study visit. Dietary intake will be assessed using 3-day food records covering 2 weekdays and 1 weekend day. Investigators will review the records during face-to-face sessions using visual aids to ensure completeness and improve portion-size estimation accuracy. Nutrient intake will be analyzed using the Malaysian Food Composition Database [29]. Physical activity will be assessed using the short-form International Physical Activity Questionnaire, available in both English and Malay, which has been validated for the Malaysian population [30,31]. Test product compliance records and adverse event reports will be obtained during each postintervention visit. Any serious adverse event or major complaint will be promptly managed by the study physician and reported to the responsible authorities within 48 hours, with appropriate medical action documented. Leftover blood and stool biospecimens from each visit will be stored at −80 °C in the institutional biobank for future research on disease biomarkers, gene expression, or microbial function, subject to ethics approval and participant consent.

### Gut Microbiota Analysis

Gut microbiota profiling will be performed using 16S rRNA gene sequencing targeting the V3-V4 region. DNA will be extracted using the Maxwell RSC system (Promega), and library preparation will follow Illumina’s 16S Metagenomic Sequencing protocol [32]. Amplicons will be barcoded, pooled equimolarly, and sequenced on the Illumina MiSeq platform (2×250 bp). Raw reads will be demultiplexed, quality-filtered, and denoised using DADA2 in QIIME 2 to generate amplicon sequence variants and remove chimeras [33,34]. Taxonomic classification will be assigned using a pretrained SILVA 138 database classifier [33]. Microbial diversity will be evaluated using α diversity indexes (Shannon, Faith phylogenetic diversity) and beta diversity metrics (Bray-Curtis, UniFrac distances) [35-38]. Differential abundance analysis will be conducted using appropriate statistical frameworks, such as ANCOM-BC or MaAsLin2, with false discovery rate correction [39]. Downstream analyses will be guided by metadata and study endpoints to identify microbial shifts associated with each intervention.

### Statistical Analysis

Clinical data will be presented as mean (SD) unless stated otherwise. Statistical analyses will be performed using SPSS Statistics (version 28.0; IBM Corp) [40]. The normality of continuous variables will be assessed using the Shapiro-Wilk test. Participants’ baseline characteristics will be summarized using descriptive statistics. Differences in glycemic responses across the 3 study arms will be assessed using either 1-way ANOVA or the Kruskal-Wallis test, depending on data distribution. Changes in clinical variables before and after the intervention will be analyzed using repeated-measures ANOVA, with time (before vs after), group (intervention arm), and the time-by-group interaction as factors. Sociodemographic characteristics, dietary intake, and physical activity levels will be considered as covariates where appropriate. A *P* value of <.05 will be considered statistically significant. Analyses will be conducted according to the intention-to-treat principle, with per-protocol analyses performed for participants who complete the study without major deviations. Missing data will be handled using appropriate imputation methods, and any exploratory analyses will be clearly identified and interpreted with caution.

## Results

Recruitment for this trial is scheduled to commence in January 2026 and is expected to conclude by September 2026. Data collection and analysis will be conducted sequentially after each intervention phase, with overall completion anticipated by March 2027. As of December 2025, no participants have been enrolled.

## Discussion

This protocol outlines a trial designed to examine the short-term effects of NNSs, specifically sucralose and saccharin, on glycemic control and gut microbiota composition among patients with T2DM. Glycemic control continues to be a central focus in diabetes management, and growing evidence suggests that the gut microbiota plays a significant role in regulating glucose metabolism [41]. Therefore, investigating these 2 domains concurrently in individuals with T2DM is both timely and clinically relevant. This study addresses a critical knowledge gap, as most prior research has focused on healthy populations, leaving limited evidence regarding metabolic and microbial responses in people with T2DM [2-4,7-13,42]. To date, only 1 study conducted in 2003 has examined the impact of sucralose consumption on glycemic control among individuals with T2DM, reporting no significant effects [43]. Among healthy participants, findings on both glycemic regulation and gut microbiota modulation following sucralose or saccharin intake have been inconsistent. Some studies have reported decreased insulin sensitivity after sucralose or saccharin consumption [2-4,10-13], whereas others have found no meaningful changes in glucose metabolism [7-9,42]. Similarly, evidence regarding gut microbiota alterations remains inconclusive, with several investigations observing significant shifts in microbial composition [2-4], while others have reported no detectable differences [7,9]. These discrepancies likely arise from variations in study design, intervention duration, dosage, sweetener formulation, and participant characteristics. Differences in demographic, dietary, and lifestyle factors also contribute to heterogeneity and make comparisons between studies challenging.

This trial aims to overcome these inconsistencies through a tightly controlled crossover design, enabling each participant to serve as their own control and thereby minimizing interindividual variability. A 1-week intervention period was selected to assess short-term effects while maintaining participant compliance and feasibility, followed by a 4-week washout period to avoid carryover effects. The selected NNS dosages represent the upper acceptable daily intake recommended in medical nutrition therapy guidelines, ensuring both safety and sufficient exposure to detect measurable outcomes [22]. Capsules will contain pure sucralose or saccharin to isolate the effects of the sweeteners themselves, excluding confounding components from commercial formulations. Recruitment through government health care facilities allows for a clinically characterized sample reflective of the local T2DM population. Restricting participation to males within a defined age and BMI range enhances phenotype consistency, while inclusion criteria based on disease duration and treatment regimen further standardize metabolic profiles. Gut microbiota profiling using 16S rRNA sequencing targeting the V3-V4 region will facilitate comparison with established microbiome literature [44].

Several limitations warrant consideration. The relatively short intervention period and modest sample size may not capture longer-term or subtle metabolic and microbial changes. Although participants will be encouraged to maintain their usual diet and physical activity, residual variability in lifestyle behaviors could influence outcomes. These confounders will be monitored using validated dietary and physical activity recall tools to aid interpretation. Furthermore, as limited baseline microbiome data are available for Malaysian individuals with T2DM, observed microbial shifts should be interpreted within the context of population-specific dietary and genetic factors. Nonetheless, the comprehensive baseline characterization and rigorous design of this study are expected to strengthen internal validity and provide a valuable foundation for future investigations.

In conclusion, this trial will generate novel evidence on short-term metabolic and microbial responses to NNSs in individuals with T2DM. Findings may help refine dietary recommendations and guide policy decisions regarding NNS consumption in Asian populations with T2DM. Beyond clinical implications, the results are expected to inform public and industry perspectives on NNS safety and contribute to the broader understanding of gut microbiome-metabolism interactions, supporting the design of future large-scale and longer-term clinical trials.

## Appendix

Multimedia Appendix 1 Peer review report by The Fundamental Research Grant Scheme (FRGS), Ministry of Higher Education Malaysia.

## Abbreviations

HOMA-IR: homeostatic model assessment for insulin resistance
NNS: nonnutritive sweetener
T2DM: type 2 diabetes mellitus
