# Supplementary material for: Short-Term Effects of Nonnutritive Sweetener (Sucralose and Saccharin) Consumption on Glycemic Control and Gut Microbiota in Patients With Type 2 Diabetes: Protocol for a Double-Blind, Randomized, Placebo-Controlled, Crossover Trial
Source: JMIR Res Protoc. 2025 Dec 10;14:e82695. doi: 10.2196/82695 (PMC12739451; doi:10.2196/82695)
Supplement: Multimedia Appendix 1 [file resprot_v14i1e82695_app1.pdf]

Peer Review Form for Research Proposal Evaluation (Faculty Level)

NO.	EVALUATION CRITERIA	COMMENTS
1	Title (5%)<br>A specific study that reflects a fundamental issue requiring resolution / demonstrates novelty<br>Concise and accurately reflects the content of the research proposal	The title seems to meet several of the criteria for the FRGS grant. The term "impact" in the title is often associated with applied or translational research, where the goal is to directly influence or improve practical outcomes, policies, or solutions. In the context of fundamental research, the focus is typically on expanding knowledge, understanding basic principles, and laying the groundwork for future applications. Suggestion: "Short-term correlation of non-nutritive sweeteners with metabolic response and gut microbiome..."
2	Executive Summary (20%)<br>Problem statement<br>Objectives<br>Methodology<br>Expected outcomes/implications<br>Significance of findings	The executive summary effectively communicates the problem, objectives, methodology, implications, and the importance of the study outcomes, providing a clear overview of the research focus and its relevance.
3	Research Background (20%)<br>Explanation of the title<br>Clear problem statement and research questions / hypotheses / theoretical framework (where applicable)<br>Incorporates citations from at least five (5) relevant and recent references	Emphasize how your research contributes to the foundational understanding of a particular phenomenon, which may have implications for applied or practical aspects in the future. The general objective you've outlined is comprehensively investigating the impact of non-nutritive sweeteners (NNSs), specifically sucralose and saccharin, on metabolic response and gut microbiota in individuals with type 2 diabetes mellitus (T2DM), which i think is more aligned with an applied or translational research scope rather than a purely fundamental research scope. If you intend to position your research as more fundamentally oriented, you might consider rephrasing the objective to highlight the

	Aligned with government policies, national agenda, and global aspirations (able to contribute to solving issues at the local, national, or global level)	investigation of basic biological processes or mechanisms, without explicitly emphasizing the practical consequences or "impact" on individuals with diabetes. For example:<br><br>"To elucidate the physiological responses and alterations in gut microbiota associated with the consumption of non-nutritive sweeteners, sucralose, and saccharin, in the context of type 2 diabetes mellitus." or "To comprehensively investigate the metabolic response and alterations in gut microbiota associated with the consumption of non-nutritive sweeteners (NNSs), specifically sucralose and saccharin, in individuals with type 2 diabetes mellitus."
4	Objectives (10%)<br><br>Specific, Measurable, Achievable, Realistic, and Time-bound (SMART)	These objectives encompass a balance between applied and fundamental research elements. They involve exploring specific physiological and molecular changes, which aligns with fundamental research goals, while also addressing practical implications for individuals with type 2 diabetes. In general, both objectives appear to be SMART, with a clear focus on specific measurements, achievability, relevance to the overall study, and, to some extent, the potential for time-bound elements. Consider adding explicit time frames if applicable, and ensure that the time aspect is clear in your study design or protocol.
5	Methodology (20%)<br><br>Clear and detailed description of methodology (may include fieldwork, sampling techniques, interviews, analyses, various laboratory phases, experimental protocols, statistical analyses	The described method leans more towards applied research, specifically clinical or intervention research. Here's why: 1. The study involves instructing participants to consume specific substances (sucralose, saccharin, or placebo) for a defined period. This kind of intervention is typical in applied research where the goal is often to observe the effects of a treatment or substance in a real-world or controlled setting. 2. The method involves precise dosing of sucralose and

	Able to achieve the stated research objectives	saccharin based on acceptable daily intake (ADI) values. This indicates a practical application of theoretical knowledge, aligning with applied research where researchers often aim to implement interventions based on established guidelines. 3. The individualization of each participant's intake based on their weight and ADI values adds a practical and personalized element to the study. This personalized approach is often a characteristic of applied research, where interventions are tailored to individual circumstances. 4. Instructing participants to register and report any adverse events adds a clinical and applied dimension to the study. Monitoring and reporting adverse events are common practices in clinical and applied research to ensure participant safety during interventions.
	Includes research design, flow chart, Gantt chart, activities, and milestones	
6	Expected Outcomes (5%)	-
	New theories or novel findings/knowledge	
	Publications in indexed (top-tier) journals / intellectual property	
	Human capital development – Master’s or PhD students	
	Generates societal, economic, and national impact	
7	Research Group Track Record (5%)	-
	Proven track record and previously successful research projects	
	Researcher qualifications	
	Balanced team composition	
8	Quality of the Research Proposal (10%)	-
	Thoroughness	

	Appropriate use of language (grammar, spelling, sentence structure)	
	Good formatting and presentation	
9	Project Criteria Elements (5%)	
	Research at the frontier of knowledge and emerging horizons, high impact, and with the potential to produce quality publications	The design and execution of the study, particularly the specific intervention and participant instructions, suggest a more applied approach. You have to ensure it is aligned with fundamental research.

		Tarikh Kuatkuasa: 01/01/2022
Universiti Kebangsaan Malaysia *The National University of Malaysia*	**BORANG PENILAIAN PROJEK PENYELIDIKAN PERINGKAT PTJ**<br>**SKIM GERAN PENYELIDIKAN FUNDAMENTAL (FRGS)**	

**Nama Ketua Projek**	**Harvinder Kaur A/P Gilcharan Singh**
**Tajuk Penyelidikan**	**Short-term impact of non-nutritive sweeteners (sucralose and saccharin) consumption on metabolic response and gut microbiome in type 2 diabetes patients**
**Domain Penyelidikan**	**Clinical and Health Sciences**
**Fakulti/Institut/Pusat**	**FSK**

SKALA PEMARKAHAN

Sangat Lemah	Lemah	Sederhana	Baik	Cemerlang
1-2	3-4	5-6	7-8	9-10

BAHAGIAN A: KRITERIA PENILAIAN

NO.	KRITERIA PENILAIAN	SKOR (1-10) (Sila isi)	MARKAH SEBENAR (%)	ULASAN
1	**Tajuk (5%)**<br>Kajian spesifik yang menggambarkan isu fundamental yang perlu diselesaikan/ menunjukkan novelti<br>Ringkas dan menggambarkan kandungan cadangan penyelidikan	**9**	**4.5**	Improvements have been made.
2	**Ringkasan Eksekutif (20%)**<br>Pernyataan masalah<br>Objektif<br>Metodologi<br>Jangkaan hasil/implikasi<br>Kepentingan hasil	**9**	**18**	The executive summary effectively communicates the problem, objectives, methodology, implications, and the importance of the study outcomes, providing a clear overview of the research focus and its relevance.
3	**Latar Belakang Kajian (20%)**<br>Huraian tajuk<br>Penyataan masalah dan persoalan kajian /hipotesis/kerangka teoritikal jelas (jika berkenaan)	**8**	**16**	Aligned with the title

3	Membuat petikan sitasi sekurang-kurangnya lima (5) rujukan terbaharu yang berkaitan<br>Sejajar dengan dasar kerajaan, agenda negara dan aspirasi global (boleh membantu mengurangkan masalah di peringkat tempatan, kebangsaan atau dunia)	8	16	Aligned with the title.
4	**Objektif (10%)**<br>Spesifik (Specific), Boleh diukur (Measurable), Boleh dicapai (Achievable), Realistik (Realistic) dan Dalam Jangka Masa (Within Time-Frame) (SMART)	**8**	**8**	Improvements have been made.
5	**Metodologi (20%)**<br>Penerangan metodologi yang jelas dan terperinci (boleh terdiri daripada kerja lapangan, persampelan teknik, sesi temu bual, analisis, kerja-kerja makmal fasa berbeza, protokol eksperimen, analisis statistik)<br>Dapat mencapai objektif penyelidikan<br>Ada reka bentuk penyelidikan, carta alir, carta gantt, aktiviti dan jejak kunci	**9**	**18**	Aligned with the objectives.
6	**Jangkaan Hasil (5%)**<br>Teori baharu atau penemuan/pengetahuan baharu<br>Penerbitan dalam jurnal berindeks (top tier)/harta intelek<br>Penghasilan bakat - Pelajar sarjana atau PhD<br>Memberi impak terhadap masyarakat, ekonomi dan negara	**9**	**4.5**	
7	***Track Record* Kumpulan Penyelidikan (5%)**<br>*Track record* dan projek penyelidikan terdahulu yang telah berjaya<br>Kelayakan penyelidik<br>Ahli kumpulan yang seimbang	**9**	**4.5**	
	**Kualiti Cadangan Penyelidikan (10%)**			

8	Teliti<br>Penggunaan bahasa yang bersesuaian (tatabahasa, ejaan, binaan ayat)<br>Format dan persembahan yang baik	**9**	**9**	
9	**Elemen Kriteria Projek (5%)**<br>Penyelidikan di perbatasan ilmu dan ambang ufuk, berimpak tinggi dan berupaya menghasilkan penerbitan yang berkualiti	**8**	**4**	Improvements have been made
	**JUMLAH**		**86.5**	

BAHAGIAN B : KRITERIA PENILAIAN TAMBAHAN

NO.	PERKARA	SKOR (1-10) (Sila isi)	MARKAH SEBENAR (%)	ULASAN
1	Carian Paten (2%)	**9**	**1.8**	ok
2	Kolaborasi (2%)	**9**	**1.8**	
3	Risiko (1%)	**8**	**0.8**	
	**JUMLAH**		**4.4**	

BAHAGIAN C : MARKAH KESELURUHAN (%)

Bahagian A	**86.5**
Bahagian B	**4.4**
**JUMLAH KESELURUHAN**	**90.9**

BAHAGIAN D : CADANGAN PERUNTUKAN

VOT	CADANGAN PERUNTUKAN (RM)
V11000 - Upah dan Elaun	50,400.00

V21000 - Perjalanan dan Pengangkutan	13,800.00
V24000 - Sewaan	
V27000 - Bekalan dan Bahan Penyelidikan	140,182.00
V28000 - Baik Pulih Kecil dan Ubahsuai	
V29000 - Perkhidmatan Ikhtisas	10,000.00
V35000 - Aksesori dan Peralatan<br>Nama alat (jika diluluskan) :<br>1 -<br>2 -	
**JUMLAH**	**214,382.00**

**BAHAGIAN E: PANEL PENILAI**	
**Nama**	Prof. Dr. Ruzita Abd. Talib
**Tandatangan**	Ruzita
**Tarikh**	24.11.2023

Rating	**Kriteria Pemarkahan (Markah Keseluruhan %)**	**Keputusan**
1	59 dan ke bawah	**Tidak disokong (79 dan ke bawah)**
2	60 – 64	
3	65 – 69	
4	70 – 74	
5	75 – 79	
6	80 – 84	**Disokong (80 – 84)**
7	85 – 89	**Sangat disokong (85 – 105)**
8	90 – 94	
9	95 – 99	
10	100 - 105	

Feedback from the Research Proposal Evaluation Committee for the Fundamental Research Grant Scheme (FRGS), Ministry of Higher Education (MOHE) Malaysia

Overall Remark

This proposal is well written and there are merits to it that justify this proposal to be recommended. However, one (with several others) minor flaw(s) that will require the researchers' attention as stated below. We hope they will take action to address and rectify the proposal to accommodate these.

Good research but the patient recruitment for this study not included for 2 years research maybe maintain the budget to rm 150,000.00

Title and Keywords: suggest to include keywords from tite

Keyword: make sure the keywords similar with the title- done

Executive Summary: acceptable but no method details included- done

Research Background: RS- maybe exclude the references and please specify on the real gap of the study which is lacking. Example used of corse sugar in food and drinks has implicated in DM 2 cases and unhealthy BMI .Therefore alternatives substitute of the corse sugar in drinks and food are really needed. This will eventually reduced DM 2 and have a more healthy nation.- done

HY- good and precise.

RQ- good and precise

LR-clear and the figures is very good

poG- relevant

References: please make sure include the publication year. done

Objective: clear and achieable

Methodology: why o.o8 %. how does the patient recruit.

what is the popultion of DM patients in that clinic. done

Flow Chart: maybe she should include ethical clearance.- done

Equipment: maybe specifiy the clinic that the patient recruit

Expected Result: OK

Expected Publication: good

Track Record and Composition of Team: comprehensive team members

Project Leader Publications: ok

Project Member: comprehensive team

Robustness of Proposal: maybe the flowchart should include study obj- done

Research Collaborator: will be good to include individual from the clinic- done

Exp Outcome Impact: PRECISE

Exp Outcome IP: maybe can specify whether copyright or IP- done

Patent Search: maybe include trend details- done

Research Activity: precise

maybe include ethical clearance- done

Risk Assessment: ok

Vot 11000: ok

Vot 27000: ok

Vot 29000: are we allow to hired a proof editor

EVALUATION OF POSTGRADUATE RESEARCH PROPOSAL
FACULTY OF HEALTH SCIENCES

**Name of Student**	**:**	**Tan Huey Shin**
**Matrix No.**	**:**	**P153672**
**Programme**	**:**	
**Specialization**	**:**	
**Supervisory committee**	**:**	**Dr. Harvinder Kaur A/P Gilcharan Singh, Dr. Nor Aini Binti Jamil @ A.Wahab, Dr. Vanitha Mariappan, Prof. Dr. Snigdha Misra, Dr. Suhaili Naim Binti Mustapa**
**Research centre**	**:**	

Item	Evaluation Criteria	Rating Score	Comments
**1.**	**Suitability of Research Title**<br><br>(Research title able to demonstrate the meaning and cover the scope of study)	4	Suitable title
**2.**	**Introduction and Literature Review**<br><br>(Suitability of Background information, problem statements and relevant literatures)	4	Up to date literature review

Item	Evaluation Criteria	Rating Score	Comments
3.	**Scientific Merit of Research Justification and Objectives**<br><br>(Research aims/objectives and justifications are well-defined and highlight the scientific merit of the study)	4	
4.	**Research Methodology**<br><br>(Suitability of research design, structure, approach and planning of research project)	4	Just a suggestion, to ensure that diet intake is maintained pre & during intervention, should diet record before intervention begins also need to be conducted?
5.	**Research Impact**<br><br>(Benefits and contributions of the research to the respective field)	5	

Item	Evaluation Criteria	Rating Score	Comments
6.	**Overall Performance**<br><br>(Presentation skills, visual aids, shows confidence during Q&A session, etc.)	4	
7	**Suitability of the specialization**<br>(if the study is within the scope of specialization.)	5	
	**Total Score**	30	

Rating Score: 1 = Very Poor, 2 = Poor, 3 = Average, 4 = Good, 5 = Excellent

Decision: **Approved (   )** **Approved with corrections (/)** **Re-write (   )**
Re-presentation (   ) **Rejected (   )**

Signature :

Name of reviewer : Dr. Nurul Huda Razalli

Date : 13/3/2025

EVALUATION OF POSTGRADUATE RESEARCH PROPOSAL
FACULTY OF HEALTH SCIENCES

**Name of Student**	**:**	**Tan Huey Shin**
**Matrix No.**	**:**	**P153672**
**Programme**	**:**	**Master of Health Science**
**Specialization**	**:**	**Dietetics**
**Supervisory committee**	**:**	**Dr. Harvinder Kaur A/P Gilcharan Singh, Dr. Nor Aini Binti Jamil @ A.Wahab (UKM) Dr. Vanitha Mariappan (UKM), Prof. Dr. Snigdha Misra (Monash University) and Dr. Suhaili Naim Binti Mustapa (Klinik Kesihatan Setapak)**
**Research centre**	**:**	**ReACH**

Item	Evaluation Criteria	Rating Score	Comments
**1.**	**Suitability of Research Title**<br><br>(Research title able to demonstrate the meaning and cover the scope of study)	**3**	You will only be studying males with T2DM so this needs to be reflected in your title.

**2.**	**Introduction and Literature Review**<br><br>(Suitability of Background information, problem statements and relevant literatures)	4	This can be further improved – perhaps the flow of your lit review as this can used for your thesis. All literature is up to date well done and you have included key papers relevant to your study.

Item	Evaluation Criteria	Rating Score	Comments
**3.**	**Scientific Merit of Research Justification and Objectives**<br><br>(Research aims/objectives and justifications are well-defined and highlight the scientific merit of the study)	4	
**4.**	**Research Methodology**<br><br>(Suitability of research design, structure, approach and planning of research project)	5	Your questionnaires were all separated – I am not sure if you would like to combine them, so it does not look like a lot for your participants. You can perhaps think of compiling in a booklet form so it easier to use for f-up etc
**5.**	**Research Impact**<br><br>(Benefits and contributions of the research to the respective field)	4	

PEJABAT TIMBALAN DEKAN (SISWAZAH), FAKULTI SAINS KESIHATAN
Universiti Kebangsaan Malaysia, Kuala Lumpur Campus,
Jalan Raja Muda Abdul Aziz, 50300 Kuala Lumpur, Wilayah Persekutuan Kuala Lumpur, Malaysia
Tel.: +603-9289 7517 / 7614 / 7983 e-mel: postgraduate.fsk@ukm.edu.my Web: www.ukm.my/fsk

Item	Evaluation Criteria	Rating Score	Comments
6.	**Overall Performance**<br><br>(Presentation skills, visual aids, shows confidence during Q&A session, etc.)	5	
7	**Suitability of the specialization**<br>(if the study is within the scope of specialization.)	5	This is a very important study, all the best
	**Total Score**	30	

Rating Score: 1 = Very Poor, 2 = Poor, 3 = Average, 4 = Good, 5 = Excellent

Decision: **Approved (/)** **Approved with corrections ( )** **Re-write ( )**
Re-presentation ( ) **Rejected ( )**

Signature : K.Ahn

Name of reviewer :Dr.Shanthi Krishnasamy Date : 11/03/25

Rumusan Pembetulan Proposal Penyelidikan / *Summary of Research Proposal Corrections*
Pls referred to the latest uploaded → updated 13 Mar revised proposal

No.	Komen penilai (*reviewers' comments)*	Pembetulan yang telah dibuat *(Corrections made in the proposal)*	Muka surat & perenggan dalam proposal di mana pembetulan telah dilakukan (*Page & paragraph number where corrections are made)*
1	Penilai 1 (*Evaluator 1)* *:Dr.Shanthi Krishnasamy*	Comment 1: You will only be studying males with T2DM so this needs to be reflected in your title.<br>Answer 1: The study title, objectives, research questions, and hypothesis have been updated to explicitly reflect that only males with T2DM will be included.<br><br>Comment 2: Your questionnaires were all separated – I am not sure if you would like to combine them, so it does not look like a lot for your participants. You can perhaps think of compiling in a booklet form so it easier to use for f-up etc.<br>Answer 2: The questionnaires will be compiled into a booklet for easier use and follow-up.<br><br>Comment 3: Why are also giving the participants a self reported form? You have a lot of questionnaires in your study - just thinking about the burden for your patients. Are they getting any inconvenience allowance?<br>Answer 3: Participants will use a self-reported form to record their daily food intake, exercise activities, compliance, and any adverse effects. Clear instructions will be provided to ensure ease of use and minimize burden. For dietary intake, participants will be encouraged to take	1. –<br><br>2. –<br><br>3. Page 11. 4.8.5 DIETARY DATA, mentioned "Participants will also be encouraged to provide food photos of meals consumed"<br><br>4. Page 8. Exclusion criteria, number 6.<br><br>5. Page 5, problem statement.<br><br>6. Page 8, Inclusion criteria, number 8.<br><br>7. Page 8. Exclusion criteria, number 8.

PUSAT KAJIAN, FAKULTI SAINS KESIHATAN
Universiti Kebangsaan Malaysia, Kampus Kuala Lumpur,
Jalan Raja Muda Abdul Aziz, 50300 Kuala Lumpur, Wilayah Persekutuan Kuala Lumpur, Malaysia
Tel.: +603-9289 7602 / 7370 E-mel: dfsk@ukm.edu.my Web: www.ukm.my/fsk

		photos, with reminders sent via WhatsApp. They will receive an RM50 honorarium for each visit.<br><br>Comment 4: If you are excluding vegetarians please include them as well in no 6.<br>Answer 4: Yes, vegetarians have been included in point number 6.<br><br>Comment 5: In your lit review perhaps you can include the prevalence of T2DM in males vs females . What are the glycemic levels among Msians as compared to guidelines.<br>Answer 5: I have incorporated the prevalence of T2DM in males and compared glycemic levels in Malaysian T2DM CPG recommendations.<br><br>Comment 6 during presentation from Dr Shanthi: Diet intake of patients who have practiced diabetes diet and normal diet would be very different, specify in your selection criteria help to ease the recruitment.<br>Answer: amended the sentence with emphasis that you suggested.<br><br>Comment 7 during presentation from Dr Shanthi: Exclude those on supplement.<br>Answer: Added the word supplement in exclusion criteria.<br><br>Comment 8 during presentation from Dr Shanthi: How will you dispose of blood samples?<br>Answer: added the highlighted sentences, protocol of blood sample disposal.	8. page 11. last para of 4.8.2 BLOOD TESTS.

2	Penilai 2 *(Evaluator 2) Dr. Nurul Huda Razalli*	Comment 9: Just a suggestion, to ensure that diet intake is maintained pre & during intervention, should diet record before intervention begins also need to be conducted? Answer 1: Yes, each intervention phase includes two visits: pre- and post-intervention. During each visit, dietary data will be collected. The first pre-intervention visit serves as the baseline, ensuring dietary intake is recorded before the intervention begins.	9: page 13 table 4.1 summary of participant' visits.
	Penilai Statistik *(Statistician)* -		

Nama Pelajar & tandatangan/*Student's name & signature*: Tan Huey Shin 13/3/2025 Huey

Nama Penyelia Utama & tandatangan/*Main Supervisor's name & signature*: 14/3/2025 Harvinder

DR. HARVINDER KAUR A/P GILCHARAN SINGH
MAHPC (DTN) 00571
Coordinator Of Master in Clinical Nutrition
Centre For Community Health Studies (ReaCH)
Faculty Of Health Sciences , Universiti Kebangsaan Malaysia , Kuala Lumpur

	UKM-JEP-BO02	No. Semakan: 02	Tarikh Kuatkuasa: 07 Disember 2023
UNIVERSITI KEBANGSAAN MALAYSIA *The National University of Malaysia*	**BORANG SARINGAN JAWATANKUASA ETIKA PENYELIDIKAN UKM** *(UKM RESEARCH ETHICS COMMITTEE REVIEW FORM)*		

**PENYELIDIK UTAMA** *PRINCIPAL INVESTIGATOR*	Harvinder Kaur A/P Gilcharan Singh
**TAJUK PROJEK** *PROJECT TITLE*	Short-term effect of non-nutritive sweeteners (sucralose and saccharin) consumption on glycaemic control and gut microbiota in type 2 diabetes patients
**JABATAN/INSTITUSI** *DEPARTMENT / INSTITUTION*	Faculty of health sciences, Centre for Community Health Studies (ReaCH)

***Sila tandakan [√] jika YA , [X] jika TIDAK dan [O] jika tidak berkaitan.**
Please Tick [√] if YES , [X] if NO and [O] if not applicable.

**No.** *No.*	**Perkara** *Items*	**Penyelidik Utama** *Principal Investigator*	**Ahli JEPUKM** *RECUKM Member*	**Komen Ahli JEP UKM** *Comments by REC Member*
1.	Tajuk yang sesuai<br>*The title is appropriate*	v	v	
2.	Adakah kajian seperti ini pernah dijalankan?<br>Jika ya,<br>a) Elemen baru dalam kajian ini ada dinyatakan<br>b) Penemuan daripada penyelidikan sebelum ini telah dinyatakan<br>Jika tidak,<br>c) Terdapat justifikasi yang mencukupi<br><br>*Has a similar study been done before?*<br>*If yes,*<br>*a)The novel elements in the present study have been stated*<br>*b)Findings from previous studies have been stated*<br>*If not,*<br>*c)There is sufficient justification*	v	v	

3.	Terapi piawai dinyatakan<br>*Standard therapy is stated*	v	0	
4.	a) Objektif utama adalah jelas<br>*Main objective is clear*	v	v	
	b) Objektif spesifik adalah jelas<br>*Specific objectives are clear*	v	v	
5.	Hipotesis adalah jelas<br>*The hypothesis(ses) is/are clear*	V	v	
6.	Populasi kajian telah dinyatakan dengan jelas<br>*The study population is clearly stated*	V	v	
7.	Pengiraan saiz sampel ditunjukkan & sesuai<br>*The sample size calculation is shown & appropriate*	V	v	
8.	Bentuk kajian telah dihuraikan dengan jelas<br>*The study design is clearly described:*<br>*Animal study/ observationals study (retrospective, pros), case-control or clinical trial*	V	v	
9.	Jenis persampelan telah dinyatakan<br>*Type of sampling mentioned: simple random, convenient, systematic, stratified*	V	v	
10.	Tempat kajian telah dinyatakan dengan jelas<br>*The study sites is/are clearly stated*	v	v	
11.	Kriteria Kemasukan dan pengecualian telah dinyatakan dan sesuai<br>*The inclusion and exclusion criteria are stated & appropriate*	v	v	
12.	Borang Soal Selidik telah divalidasi (untuk semua versi yang digunakan)<br>*The questionnaire(s) has (have) been validated (for all versions used)*	v	v	
13.	Carta alir kaedah kajian telah disertakan<br>*A flow chart of the study methodology is enclosed*	V	v	

14	Carta Gannt telah disediakan<br>*A Gannt chart is enclosed*	V	v	
15.	Bajet munasabah telah disertakan<br>*Reasonable budget is enclosed*	V	v	
16.	Borang Maklumat Subjek mudah untuk difahami dan mengikut format JEPUKM<br>*The subject information sheet is easy to understand and follows the RECUKM format*	v	x	1. Please simplify the methodology of research in the PIS (too lenghty and complex for layman's understanding)<br>2. There is no instruction given in PIS on what to do with the sweetener given. Should they mixed in their usual drinks and refrain from white sugar intake? Or put into food? Or consume with water directly?<br><br>3. Suggest to amend the sentence to "you will be given RM 50 for each of the study visits **to cover for travel expenses**"
17.	Borang Keizinan jelas dan mengikut format JEPUKM<br>*The consent form is clear and follows the RECUKM format*	v	v	
18.	Borang Persetujuan bagi umur kurang dari 18 tahun<br>*The assent form for less than 18 years old*	O	0	
19.	Borang konflik kepentingan telah diisi.<br>*The conflict-of-interest form had been filled and signed.*	V	v	
20.	Kebenaran/Keizinan telah diperolehi (jika perlu) daripada<br>*Approvals/ Permissions have been obtained (if required) from*<br>a) pemilik semua borang soalselidik dan sistem skor<br>*Owners of all questionnaires and scoring systems*<br><br>b) pihak berkuasa yang berkaitan, cth institusi bagi penyelidik bersama, kementerian, dsb<br>*other relevant authorities, eg institution of co-investigators, ministries, etc*<br><br>c) DCA (*Drug Control Authority)*	O	x	1. The study use "International physical activity questionaire (IPAQ)". Please find out if approval from the original owners of IPAQ is needed to use this questionaire.<br>2. Approval need to be sought from the relevant authority (ie Pejabat Kesihatan Negeri/ daerah). Please show letter of approval from the authorities.<br>3. As the research is conducted in KKM Health Clinic, please also apply NMMR approval.

21.	Isu-isu etika utama dalam kajian (jika ada)<br>*Major ethical issues in the study (if any)*	O	x	No major ethical issues.
22.	Nama-nama penyelidik bersama telah dinyatakan dengan jelas & sesuai<br>*The names of the co-investigators are clearly stated & appropriate*	v	v	
	Komen Penyaring (jika ada):<br>*Reviever comment (if any):*			

Keputusan Ahli JEPUKM:

Lulus [ ]
Approve

Pinda & Hantar Semula [v]
Revise & Resubmit

Pinda & Bentang [ ]
Revise & Present

Sebab perlu bentang
Reason(s) for the need to present

[ ] a clinical trial
[ ] storing genetic/ DNA samples for analyses
[ ] vulnerable subjects, eg mentally retarded, psychiatric pts, orphans, terminally ill etc....
[ ] proposal is very complicated and difficult to understand
[ ] other reasons (please state)………………………………………………… ....
……………………………………………………………………………………………

Nama ahli JEP, UKM : **AP Dr Suria Hayati Md Pauzi**
Name of RECUKM member
Tarikh : **16/04/25**
Date

Keputusan Pengerusi JEPUKM:

Lulus [ ]
Approve

Pinda & Hantar Semula [/]
Revise & Resubmit

Pinda & Bentang [ ]
Revise & Present

Sebab perlu bentang
Reason(s) for the need to present

[ ] a clinical trial
[ ] storing genetic/ DNA samples for analyses
[ ] vulnerable subjects, eg mentally retarded, psychiatric pts, orphans, terminally ill etc....
[ ] proposal is very complicated and difficult to understand
[ ] other reasons (please state)………………………………………………… ....
……………………………………………………………………………………………

Komen Pengerusi JEP UKM :
Please obtain approval to use the questionnaire, as well as approval from the district or state health department. MREC approval is also required.

Nama Pengerusi JEPUKM :
Name of RECUKM Chairman
Tarikh :
Date

: **PROF MADYA DR CARYN CHAN MEI HSIEN**
TIMBALAN PENGERUSI IV

01/5/2025

DOKUMEN INI ADALAH CETAKAN KOMPUTER DAN TIDAK MEMERLUKAN TANDATANGAN

9th May 2025

Pengerusi
Jawatankuasa Etika Penyelidikan UKM
Tingkat 1, Blok Klinikal, Hospital Canselor Tuanku Muhriz, Pusat Perubatan UKM,
Jalan Yaakob Latiff, Bandar Tun Razak,
56000 Cheras, Kuala Lumpur.

YBhg. Tan Sri/Dato'/Datin/Tuan/Puan,

Tajuk Penyelidikan Project Title: Short-term effect of non-nutritive sweeteners (sucralose and saccharin) consumption on glycaemic control and gut microbiota in type 2 diabetes patients

No. Rujukan Etika Ethics reference code: JEP-2025-355

Berikutan maklumbalas yang diberikan oleh pihak etika, saya telah melakukan pembetulan seperti di bawah:
As commented by the committee members, I made correction as below:

Bil.	Komen penilai dalam borang BO02	Pembetulan yang telah dibuat	Muka surat & perenggan dalam proposal di mana pembetulan telah dilakukan
1	1. The study use "International physical activity questionaire (IPAQ)". Please find out if approval from the original owners of IPAQ is needed to use this questionnaire.	According to the IPAQ FAQ, the International Physical Activity Questionnaire (IPAQ) is available under the Creative Commons Attribution 4.0 International (CC BY 4.0) license. This license permits you to share, adapt, and use the questionnaire, even for commercial purposes, as long as you provide appropriate credit, include a link to the license, and indicate if changes	12 (Proposal)

		were made.<br>https://sites.google.com/view/ipaq<br><br>I have addressed the license from IPAQ authority in Proposal in page 12.	
2	1. Please simplify the methodology of research in the PIS (too lenghty and complex for layman's understanding)<br>2. There is no instruction given in PIS on what to do with the sweetener given. Should they mixed in their usual drinks and refrain from white sugar intake? Or put into food? Or consume with water directly?<br>3. Suggest to amend the sentence to "you will be given RM 50 for each of the study visits to cover for travel expenses"	1. I have simplified the PIS for both English and Malay versions.<br><br>2. Page 2, under the "About the Test Products & Placebo", instruction is stated.<br><br>3. Page 4, under "Are There Any Costs to Participate?", amended the sentence.	Page 2 & 4 (PIS English version)<br><br>Malay version was amended accordingly in Page 3 and 5.
3.	Approval needs to be sought from the relevant authority (ie Pejabat Kesihatan Negeri/ daerah). Please show letter of approval from the authorities.	According to the protocol, the approval letter from the Pejabat Kesihatan Negeri will be applied for after obtaining ethics approval from the NMRR. However, I have attached a letter of collaboration approval from the physician in charge at the Klinik Kesihatan Setapak study site for your reference.<br><br>Currently, the NMRR ethics application is still pending full board review by the MREC. We will proceed with the	KK Setapak letter of collobration.

		necessary applications and provide updates once the approval is granted.	
4.	As the research is conducted in KKM Health Clinic, please also apply NMMR approval.	As mentioned above, the NMRR ethics approval is still pending full board review by the MREC. The necessary approvals will be updated once it is received.	N/A

Pihak kami berharap pindaan permohonan etika ini mendapat pertimbangan daripada Pengerusi Jawatankuasa Etika Penyelidikan UKM.

Yang Benar,

DR. HARVINDER KAUR A/P GILCHARAN SINGH
MAHPC (DTN) 00571
Coordinator Of Master In Clinical Nutrition
Centre For Community Health Studies (ReaCH)
Faculty Of Health Sciences , Universiti Kebangsaan Malaysia , Kuala Lumpur

Dr. Harvinder Kaur A/P Gilcharan Singh Principal Investigator
Dietetics Program | Centre for Community Health Studies
(ReaCH) Faculty of Health Sciences, Universiti
Kebangsaan Malaysia
Jalan Raja Muda Abd Aziz, 50300,
Kuala Lumpur Email:
harvinder_kaur@ukm.edu.my

Professor Dr. Mohd Shahrir Mohamed Said
Pengerusi *(Chairman)*
Jawatankuasa Etika Penyelidikan UKM
(Research Ethics Committee UKM)

Tarikh 24th June 2025
(Date)

Tajuk Penyelidikan: Short-term effect of non-nutritive sweeteners (sucralose and saccharin) consumption on glycaemic control and gut microbiota in type 2 diabetes patients
(Project Title)
No. Rujukan Etika: JEP-2025-322
(Ethics reference code)

Tarikh Pembentangan: 12 June 2025
(Date of Present)

Berikutan maklumbalas yang diberikan oleh pihak etika, saya telah melakukan pembetulan seperti di bawah:
As commented by the committee members, I made correction as below:

Bil. (No.)	Komen dalam Petikan Minit Mesyuarat *[comments in the Minute of Meeting (MOM)]*	Pembetulan yang telah dibuat *(Corrections made in the proposal)*	Muka surat & perenggan dalam proposal di mana pembetulan telah dilakukan *(Page & paragraph number where corrections are made)*
1	To include the total volume of blood in the PIS.	Added "A blood sample (~15 mL, about one tablespoon) will be taken for blood glucose and basic blood profile analysis."	Pg 3 of PIS
2	Declare that the blood will be discarded after the study.	Added "Following laboratory analysis, all samples will be disposed of in accordance with biosafety protocols, which include chemical disinfection or autoclaving, followed by disposal in compliance with the Ministry of Health Malaysia and institutional biohazardous waste guidelines."	Pg 11 of proposal study protocol
3.	To take consent for blood storage, pls adhere it according to UMBI format.	All relevant information regarding biospecimen collection and archived storage has been included in both the study protocol and optional the Participant Information Sheet (PIS). Biospecimens will be stored at an external collaborating research laboratory, we have adopted the content and ethical standards in line with UMBI's informed	Can refer to "Optional PIS & ICF" form, I have clearly stated the purpose of archiving sample.<br><br>Added archiving statement in my

		consent format to ensure compliance. The archiving of specimens is addressed in the consent process, with voluntary participation and the right to withdraw emphasized. NMRR registration and ethical clearance for biobanking will also be obtained accordingly.	protocol proposal page 11 for blood (4.8.2) and page 13 (general statement, 4.9 & 4.10).

Pihak kami berharap pindaan permohonan etika ini mendapat pertimbangan daripada pihak YBhg. Prof. Dr.
I hope you will consider my application for the proposal.

Yang benar,
(Your sincerely,)

Harvinder

Dr. Harvinder Kaur A/P Gilcharan Singh Principal Investigator
Dietetics Program | Centre for Community Health Studies (ReaCH) Faculty of Health Sciences, Universiti Kebangsaan Malaysia
Jalan Raja Muda Abd Aziz, 50300,Kuala Lumpur
Email: harvinder_kaur@ukm.edu.my
